# Caffeine, Coffee, Tea and Risk of Rheumatoid Arthritis: Systematic Review and Dose-Response Meta-analysis of Prospective Cohort Studies

**DOI:** 10.3389/fnut.2022.822557

**Published:** 2022-02-10

**Authors:** Farzaneh Asoudeh, Fatemeh Dashti, Ahmad Jayedi, Amirhossein Hemmati, Abdulmannan Fadel, Hamed Mohammadi

**Affiliations:** ^1^Department of Clinical Nutrition, School of Nutritional Sciences and Dietetics, Tehran University of Medical Sciences, Tehran, Iran; ^2^Social Determinant of Health Research Center, Semnan University of Medical Sciences, Semnan, Iran; ^3^School of Sport and Exercise Sciences, Liverpool John Moores University, Liverpool, United Kingdom

**Keywords:** rheumatoid arthritis, coffee, tea, caffeine, meta-analysis

## Abstract

**Objective:**

Prospective cohort studies on coffee, tea and caffeine in relation to the risk of rheumatoid arthritis (RA) have shown conflicting results. The aim of this study was to conduct a dose–response meta-analysis of cohort studies on the association between dietary caffeine, different types of coffee and tea consumption and the risk of RA.

**Methods:**

PubMed/Medline, Scopus and EMBASE were searched up to July 2021 to identify relevant studies that had considered different types of coffee (caffeinated or decaffeinated), tea or caffeine exposure with RA as the main, or one of the, outcome(s). Two authors independently screened 742 publications. Finally, five prospective cohort studies were included in our meta-analysis. Pooled relative risks (RRs) were calculated by using a fixed-effects model. We also performed linear and non-linear dose-response analyses to examine the dose-response relations.

**Results:**

Comparing extreme categories, we found a positive, significant association between coffee (RR: 1.30; 95% CI: 1.04–1.62; *I*^2^ = 0%, *n* = 5) and decaffeinated coffee (RR: 1.89; 95% CI: 1.35–2.65; *I*^2^ = 38.1%, *n* =3) consumption and risk of RA. One additional cup of coffee consumed per day was associated with an increased risk of RA by 6% (95% CI: 1.02–1.10; *I*^2^ = 0%). This increase in the risk of RA for one cup/d of decaffeinated coffee was 11% (95% CI: 1.05–1.18; *I*^2^ = 38). No significant association was observed between caffeinated coffee, tea or caffeine intake and the risk of RA.

**Conclusion:**

We found that a higher intake of coffee and decaffeinated coffee was associated with increased risk of RA. No significant association between caffeinated coffee, tea or caffeine intake and the risk of RA was observed.

**Systematic Review Registration:**

https://www.crd.york.ac.uk/prospero/display_record.php?RecordID=227665, identifier: CRD42021227665.

## Introduction

Rheumatoid arthritis (RA) is a prevalent autoimmune disease diagnosed by joint function deterioration, erosion of cartilage and bone distraction, which can lead to daily activity dysfunction and decreased quality of life (1). This condition may increase the risk of cardiovascular disease, cancer, and respiratory complications (2). The incidence rate of RA in industrialized countries is 5–10 per 1,000 persons (3). Patients who suffer from RA may experience symptoms including stiffness in the joints, puffy hands, weakness, fever and fatigue (4, 5).

The etiology of RA is not clear; nevertheless, studies have demonstrated that genetic and environmental factors are the most common determining factors in the pathogenesis of RA (6). Environmental factors such as cigarette smoking, air pollution, occupational exposure, as well as dietary factors, are aligned with RA development (7). Among dietary factors, intake of red meat and alcohol consumption are positively associated with the risk of RA (8, 9), while higher consumption of fruits, vegetables, fish, as well as antioxidants, are inversely associated with the risk (10). Recently, the antioxidant properties of tea and coffee and their relation to the progression of RA has been taken into consideration (11–13). Tea and coffee are rich in biologically active components including catechins, theaflavins, caffeine and chlorogenic acid (CGA), which have anti-inflammatory effects and exert protection against chronic inflammatory disease (14, 15). A recent umbrella review have demonstrated the beneficial health effects of coffee, caffeine and antioxidant properties of coffee on various health outcomes including cancers, liver health and cardiovascular disease (16). Also, in that study, no association was found between coffee as well as decaffeinated coffee consumption and risk of RA (16). Similarly, another umbrella review investigated that usual levels of tea intake reduced diverse health outcomes (17). While, in relation to RA, no association was found between tea consumption and RA (17). Overall, there are limited studies about the association between tea as well as coffee consumption and odds of RA. In this context, some of case-control studies have reported that tea consumption are related to RA development (18, 19). While with regards to coffee consumption, a case-control study showed higher coffee consumption was associated with greater risk of RA (20). A previous meta-analysis of two case-control and three cohort studies in 2014 (21) showed a significant association between coffee intake and the increased risk of RA in case-control studies, but no association was found in cohort studies. Moreover, there was no significant association for tea intake and RA incidence. The results of the previous meta-analysis were accompanied by some limitations, as they missed one study (21), and also no dose-response meta-analysis was performed. Furthermore, there was no evaluation of the type of coffee such as whether it was caffeinated or decaffeinated. Therefore, to provide updated evidence regarding the potential association of coffee and tea intake with RA risk, the present systematic review and dose-response meta-analysis of prospective cohort studies examined the association of coffee (caffeinated and decaffeinated), caffeine or tea consumption with the risk of RA in the general population.

## Methods

We reported the current meta-analysis by following the Meta-analysis of Observational Studies in Epidemiology (MOOSE) guidelines (22).

### Search Strategy

The study protocol is available at https://www.crd.york.ac.uk/PROSPERO (registration number CRD 42021227665). A systematic search of available studies was performed up to July 2021 from the online databases of PubMed/Medline, EMBASE and Scopus. The following text and MeSH heading search key words were used in our search strategy: (“Arthritis, Rheumatoid” OR rheumatoid OR “rheumatoid arthritis”) AND (“Camellia sinensis”) OR “Tea” OR “Coffee” OR “caffeine” OR “caffeinated”). There was no language or time restriction. We did not include published abstracts in the present systematic review and meta-analysis. More information related to search strategy are presented in Supplementary Table 1. References from included studies and previous reviews were also examined to avoid missing any publications.

### Inclusion and Exclusion Criteria

The title and abstract of each study were reviewed by two independent authors (F. A and F. D) to identify potentially relevant studies. Studies with the following criteria were included: (i) all human prospective cohort studies that considered coffee, tea, caffeine or types of coffee (caffeinated or decaffeinated) as the exposure and RA as the main or one of the secondary outcomes; (ii) publications in which effect sizes were reported in the form of odds ratio, rate or risk ratios, relative risk (RRs), or hazard ratios. Letters, comments, reviews, meta-analyses, ecological studies, animal studies and studies that were conducted on children, were excluded from our systematic review and meta-analysis. The detailed information of PICOS (populations, interventions or exposures, comparators, outcomes, and study designs) for inclusion and exclusion of studies is available at Supplementary Table 2.

### Data Extraction

Two independent investigators (F. A and F. D) performed the literature research and data extraction, including the name of the first author, publication year, country, age range, sex, follow-up duration, number of cases or cohort size, exposure type, exposure assessment method, outcome assessment, comparison categories, relevant adjusted effect sizes with 95% confidence intervals and confounding variables adjusted for in the statistical analysis. When eligible studies reported several risk estimates, we extracted the fully adjusted effect sizes. Disagreements were resolved through discussion by third author (HM).

### Data Synthesis and Analysis

We considered the RRs and their 95% CI as the effect size for reporting the results of the present meta-analysis. The reported HRs were considered equal to RRs (23). We calculated the highest vs. lowest estimates for the main analyses. Due to the low number of studies (*n* ≤ 5), a fixed-effects meta-analysis was performed for combining study-specific results (24), using maximally adjusted RRs with 95% CIs (25). The overall effect size was calculated with consideration of between-study heterogeneity. Cochrane's *Q*-test and *I*^2^ (26) were used as indicators of between-study heterogeneity. *I*^2^ values >50% were considered as significant heterogeneity among studies (26). Sensitivity analysis was conducted to find which particular study or group of studies affected the overall result by sequential exclusion of each study at a time. Publication bias was examined by visual inspection of funnel plot asymmetry and then formally assessed by Egger's asymmetry test (27) and Begg's test (28).

Linear dose–response meta-analysis was also performed to estimate the RRs per increment of one cup/d of coffee, caffeinated coffee, decaffeinated coffee and tea consumption, as well as per 200-mg/d increment of caffeine intake according to previous meta-analyses (29, 30), by using generalized the least squares trend estimation method (31, 32). Total number of subjects, number of cases or person-years and median intake for each level of the exposure were needed for the generalized least squares trend estimation method. For the studies that reported a range of coffee, caffeinated coffee, decaffeinated coffee, tea and caffeine intake, the midpoint of the upper and lower limits in each category was estimated as the corresponding dose. When the highest and lowest categories were open-ended, we assumed the length of these open-ended intervals to be equal to the adjacent intervals. To examine the potential non-linear dose–response associations, we used random effects dose-response meta-analysis through restricted cubic splines with three knots at 10, 50, and 90% percentiles of the distribution (33). *P*-values for non-linearity were estimated by testing the null hypothesis, in which the coefficient of the second spline was equal to zero. All statistical analyses were performed using Stata software version 14 (Stata Corp, College Station, Texas, USA) and a *p*-value < 0.05 was considered statistically significant.

### Quality Assessment of Studies

The Newcastle-Ottawa Scale (NOS) was used to evaluate the quality of the included studies (34). With the NOS method, a maximum score of nine could be assigned to a study. In the present analysis, quality scores greater than six indicated high quality studies and scores of six or less indicated low-quality studies. We evaluated the overall quality of the evidence by the NutriGrade score (35). That is a tool to judge the quality of evidence presented by the meta-analysis of cohort studies and randomized controlled trials in nutrition research (36, 37). This score includes eight components including (1) risk of bias; (2) precision of the estimate; (3) heterogeneity; (4) directness; (5) publication bias; (6) funding bias; (7) effect size; and (8) dose–response association. Total scores ranged from 0 to 10. The measurement score was estimated as follows: very low (0 to <4 points), low (4 to <6 points), moderate (6 to <8 points) or high (8–10 points) certainty of the evidence.

## Results

### Findings From the Systematic Review

**Figure 1:**
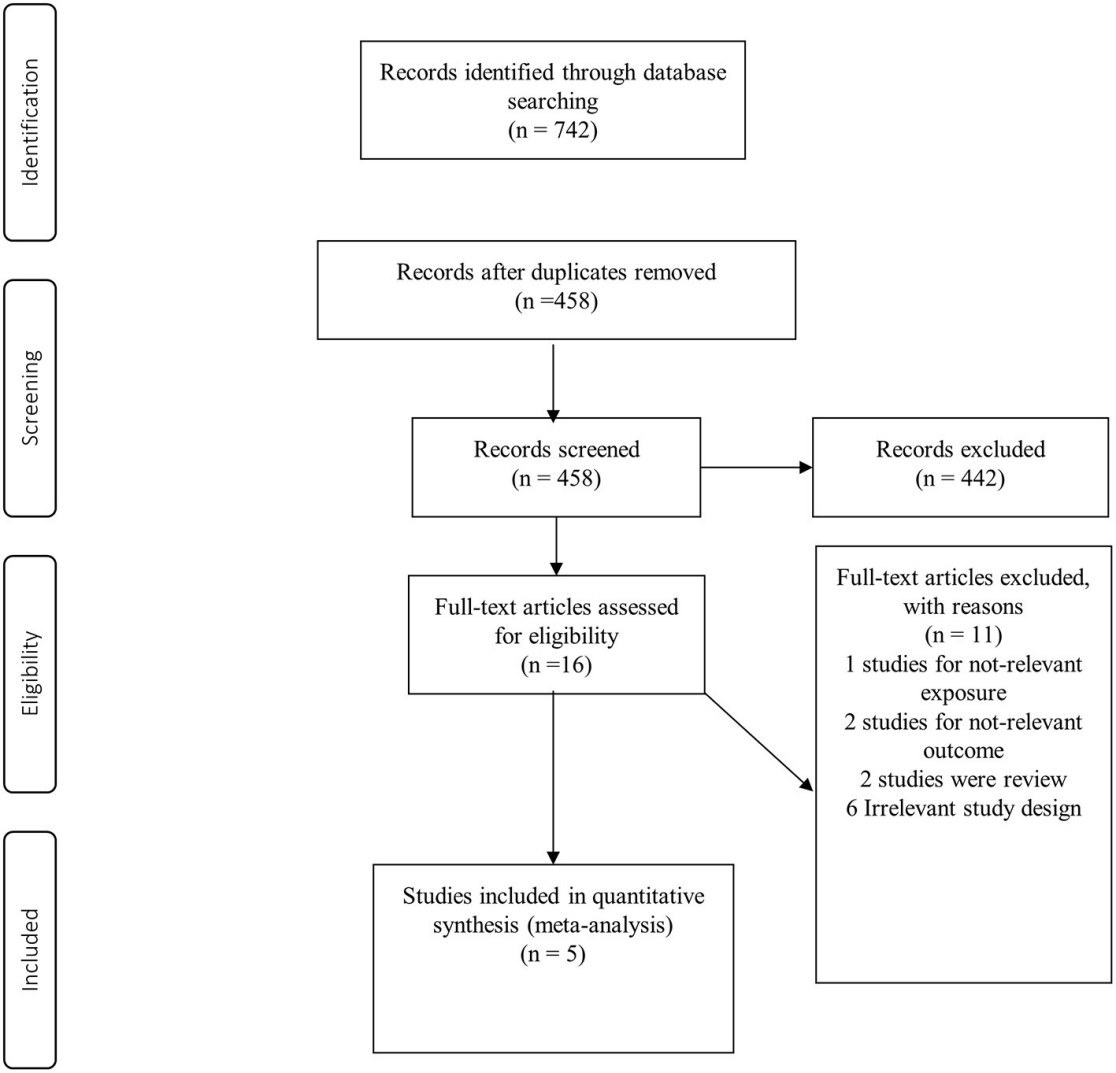
Study selection process.

Of 742 records identified, 284 studies were duplicates and 442 studies were excluded during the screening of title and abstract (Figure 1). After reading the full texts of the remaining studies, 11 studies were excluded for the following reasons: the relevant exposure of interest was not reported (*n* = 1), the study outcome was not relevant (*n* = 2), review articles (*n* = 2), and irrelevant study design (*n* = 6) (Supplementary Table 3). Finally, five prospective cohort studies (38–42) with a total of 266,985 participants were included for meta-analysis. The characteristics of the included studies are shown in Table 1. Total number of cases with RA were 1,018, varying from 69 to 480 in these studies. The studies were conducted from 2000 to 2019; three from the USA (39–41), one from Demark (42) and one from Finland (38). Age at the beginning of the studies ranged from 20 to 98 years, and the median duration of follow-up ranged from 5.3 to 19 years. In terms of exposure assessment, four studies had used food frequency questionnaires (39–42) and one study used a self-questionnaire and interview for the exposure assessment (38). All the included studies were of high quality, based on the NOS. Quality assessment of the studies is shown in Table 1.

**Table 1:** Characteristics of included prospective cohort studies.

**References, country**	**Age, gender**	**Follow up**	**Cases/ cohort size**	**Exposure assessment (items)**	**Research study**	**Exposure type**	**Outcome assessment**	**Contrast**	**RR (95% CI), highest versus lowest**	**Covariates**	**NOS**
Lamichhane et al. (40), USA	50–79 women	6 y	185/76,853	FFQ	WHI-OS	Coffee Tea Caffeine Caffeinated coffee Decaffeinated coffee	Self-reported RA and DMARD	≥4 cups/d vs. none ≥4 cups/d vs. none ≥242.96 vs. ≤ 57.53 mg/d ≥4 cups/d vs. none ≥4 cups/d vs. none	1.29 (0.84–1.98) 1.78 (0.83–3.82) 1.83 (1.07–3.15) 1.37 (0.84–2.23) 1.76 (0.92–3.36)	Age, race/ethnicity, marital status, smoking history, alcohol use, use of hormone therapy, education, BMI	8
Pedersen et al. (42), Denmark	50–64 both	5.3 y	69/56,691	FFQ	DNPR	Coffee	ACR criteria	per 200 g/d	1.10 (0.99–1.21)	Age, gender, tobacco smoking, education	8
Mikuls et al. (41), USA	55-69 women	12 y	158/31,336	FFQ (127)	IWHS	Coffee Tea Caffeine Caffeinated coffee Decaffeinated coffee	ACR criteria	≥4 cups/d vs. none >3 cups/d vs. none >376.5 vs. <29.1 mg/d ≥4 cups/d vs. none ≥4 cups/d v.s none	1.56 (0.80–3.06) 0.35 (0.13–0.97) 0.94 (0.58–1.52) 0.98 (0.60–1.61) 2.44 (1.52-3.89)	Age, marital status, smoking history, alcohol use, age at menopause, and use of hormone replacement therapy	8
Karlson et al. (39), USA	34–59 women	19 y	480/83,124	FFQ	NHS	Coffee Tea Caffeine Caffeinated coffee Decaffeinated coffee	ACR criteria	≥4 vs. 0 cups/d >3 vs. 0 cups/d >700 vs. <142 mg/d ≥4 vs. 0 cups/d ≥4 vs. 0 cups/d	1.2 (0.9–1.7) (0.7–1.8) (0.8–1.4) (0.8–1.6) 1.1 (0.5–2.2)	Age, alcohol use, smoking, BMI, age at menarche, age at first birth, parity, breastfeeding, oral contraceptive use, use of hormone replacement therapy	6
Heliövaara et al. (38), Finland	20–98 both	17 y	126/18,981	Self-questionnaire and interview	MCHES	Coffee	Record linkage Medical record	10 vs. 0 cups/d	3.95 (0.89–17.51)	Age, sex	9

*FFQ, food frequency questionnaire; RA, rheumatoid arthritis; DMARD, disease-modifying anti-rheumatic drugs; BMI, body mass index; ACR, American College of Rheumatology; NOS, Newcastle-Ottawa Scale; WHI-OS, Women's Health Initiative Observational Study; DNPR, Danish National Patient Registry; IWHS, Iowa Women's Health; NHS, Nurses' Health Study; MCHES, Mobile Clinic Health Examination Survey Study*.

### Findings From the Meta-analysis

#### Association Between Coffee Consumption and Risk of RA

**Figure 2:**
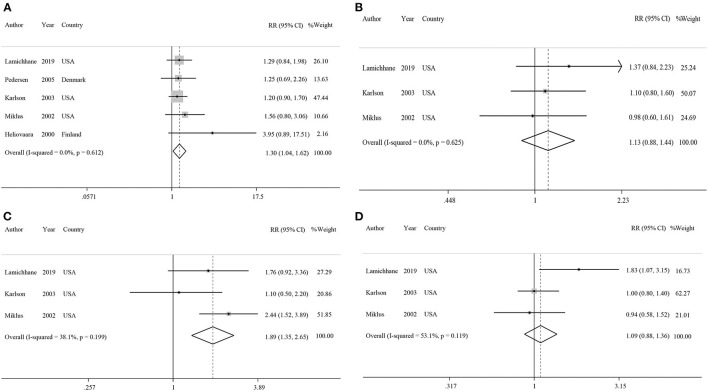
Forest plots showing relative risks of developing rheumatoid arthritis for highest vs. lowest categories of **(A)** coffee intake, **(B)** caffeinated coffee intake, **(C)** decaffeinated coffee intake, and **(D)** caffeine intake.

**Figure 3:**
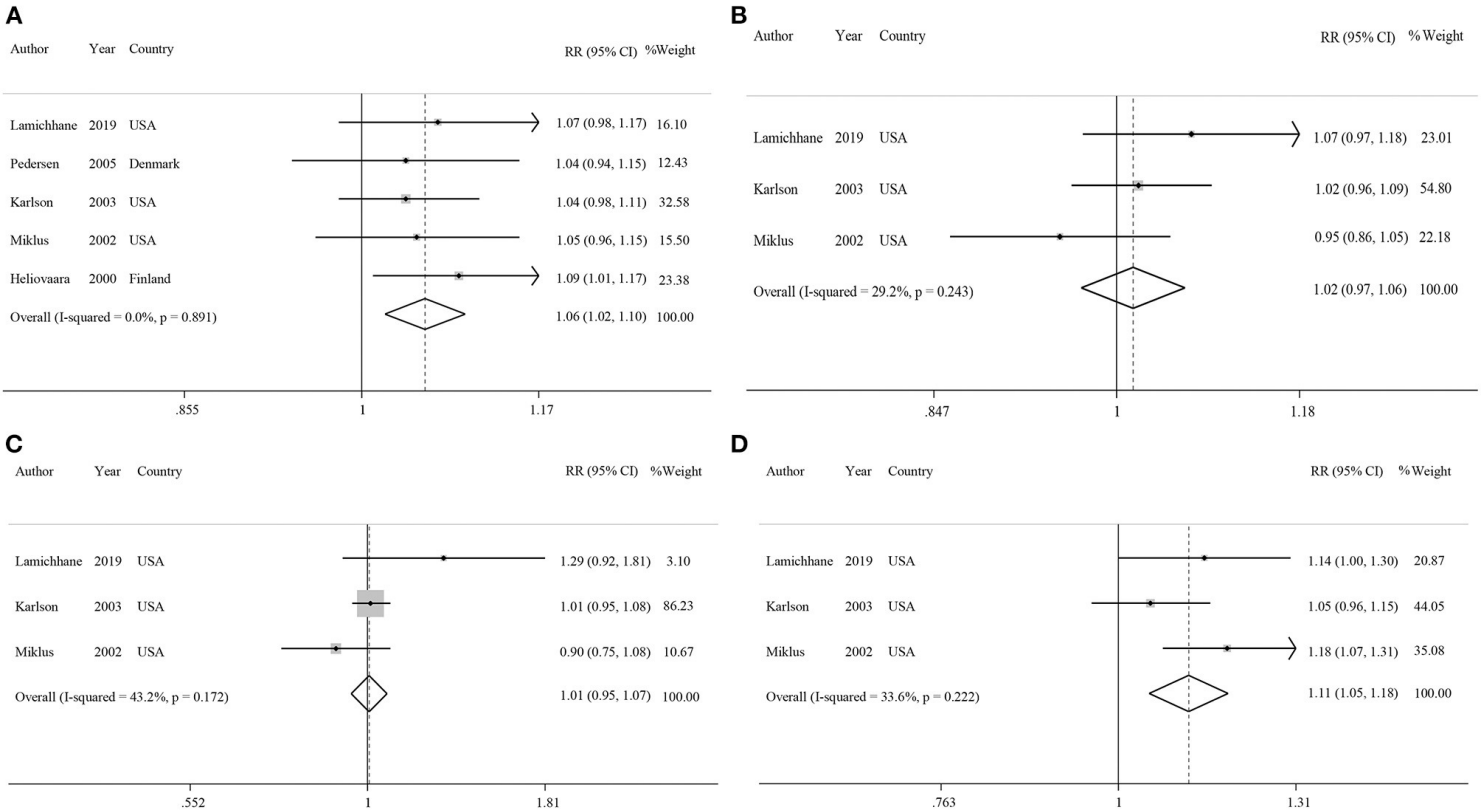
Forest plots showing linear dose-response associations for each one cup/day increase of **(A)** coffee intake, **(B)** caffeinated coffee intake, **(C)** decaffeinated coffee intake, and **(D)** each 200 mg/d increase of caffeine intake with risk of rheumatoid arthritis.

**Figure 4:**
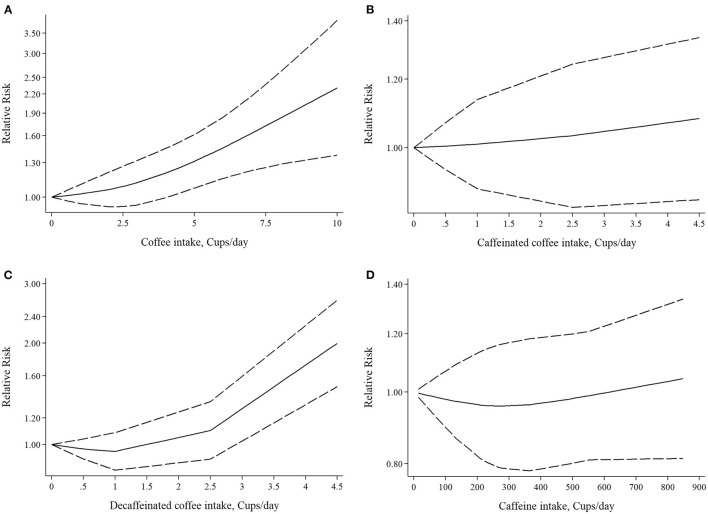
Nonlinear dose-response associations of **(A)** coffee intake, **(B)** caffeinated coffee intake, **(C)** decaffeinated coffee intake, **(D)** caffeine intake and risk of rheumatoid arthritis.

Five studies had examined the association between coffee intake and subsequent risk of RA (38–42). Pooled effect sizes for the highest vs. lowest category of coffee intake indicated a positive statistically significant association with risk of RA (RR: 1.30; 95% CI: 1.04–1.62; Figure 2A). There was no statistically significant between-study heterogeneity (*I*^2^= 0.0%; *P* = 0.61). Sensitivity analysis showed that removing each particular study at a time, did not affect the summary effects. We observed some evidence of publication bias using Begg's test (*P* = 0.05) and Egger's test (*P* = 0.02). There was a significant positive association such that an additional cup of coffee per day was correlated with a 6% increase in the risk of RA (RR: 1.06; 95% CI: 1.02–1.10; Figure 3A). Non-linear dose response analysis showed a positive monotonic relationship between coffee intake and risk of RA (P_non−linearity_ = <0.01; Figure 4A). The quality of the evidence was rated as moderate based on the NutriGrade score (Supplementary Table 4).

#### Association Between Caffeinated Coffee Consumption and Risk of RA

Three studies reported on the association between caffeinated coffee intake and subsequent risk of RA (39–41). Pooled effect sizes of the highest vs. lowest category of caffeinated coffee intake showed no significant association with the risk of RA (RR: 1.13; 95% CI: 0.88–1.44), without evidence of between-study heterogeneity (*I*^2^= 0%; *P* = 0.62; Figure 2B). In sensitivity analysis, we found that no study affected the overall effects. Also, there was no evidence of publication bias by Begg's test (*P* = 0.60) and Egger's test (*P* = 0.87). There was no significant association between one cup of caffeinated coffee in a day and risk of RA (RR: 1.02; 95% CI: 0.97–1.06; Figure 3B). Non-linear dose response analysis showed no significant relationship between caffeinated coffee intake and risk of RA (P_non−linearity_ = 0.78; Figure 4B). On the basis of the NutriGrade score, the quality of the evidence was rated as low (Supplementary Table 4).

#### Association Between Decaffeinated Coffee Consumption and Risk of RA

Three studies assessed the relationship between decaffeinated coffee intake and subsequent risk of RA (39–41). Pooled summary estimates for the highest vs. lowest category of decaffeinated coffee intake showed a positive association with the risk of RA (RR: 1.89; 95% CI: 1.35–2.65; Figure 2C). We observed low evidence of between-study heterogeneity (*I*^2^ = 38.1%, *P* = 0.19). Sensitivity analysis showed that removal of the study by Mikuls et al. changed the significant summary effects to non-significant (RR: 1.43; 95% CI: 0.88–2.34). There was no evidence of publication bias by Begg's test (*P* = 0.18) and Egger's test (*P* = 0.17). We found a positive association between one cup of decaffeinated coffee and risk of RA. Each additional cup of decaffeinated coffee per day was associated with an 11% increased risk of RA (RR: 1.11; 95% CI: 1.05–1.18; Figure 3C). Non-linear dose response analysis revealed a significant, positive relationship between decaffeinated coffee intake and risk of RA (P_non−linearity_ = <0.001; Figure 4C). The quality of the evidence was rated as moderate based on the NutriGrade score (Supplementary Table 4).

#### Association Between Caffeine Consumption and Risk of RA

Three studies reported on the association between caffeine intake and subsequent risk of RA (39–41). Combining effect sizes for the highest vs. lowest category of caffeine intake showed no significant association with the risk of RA (RR: 1.09; 95% CI: 0.88–1.36; Figure 2D). There was low evidence of heterogeneity among the studies (*I*^2^ = 43.2%; *P* = 0.17). Sensitivity analysis indicated that exclusion of any particular study from the analysis did not alter the pooled effect sizes. No publication bias was found based on Begg's test (*P* = 0.12) and Egger's test (*P* = 0.58). There was no significant association between a 200 mg increment of caffeine intake in a day and the risk of RA (RR: 1.01; 95% CI: 0.95–1.07; Figure 3D). Also, non-linear dose response analysis showed no significant relationship (P_non−linearity_ = 0.77; Figure 4D). Based on the NutriGrade score, the quality of the evidence was rated as low (Supplementary Table 4).

#### Association Between Tea Consumption and Risk of RA

**Figure 5:**
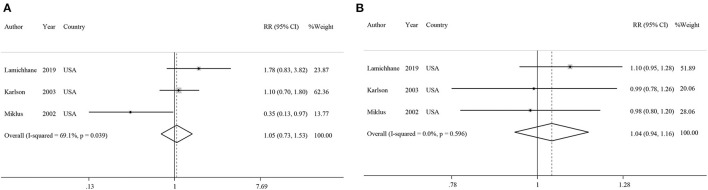
Forest plots showing relative risks of developing rheumatoid arthritis for **(A)** highest vs. lowest categories of tea intake and **(B)** linear dose-response association for each one cup/day increase of tea intake.

**Figure 6:**
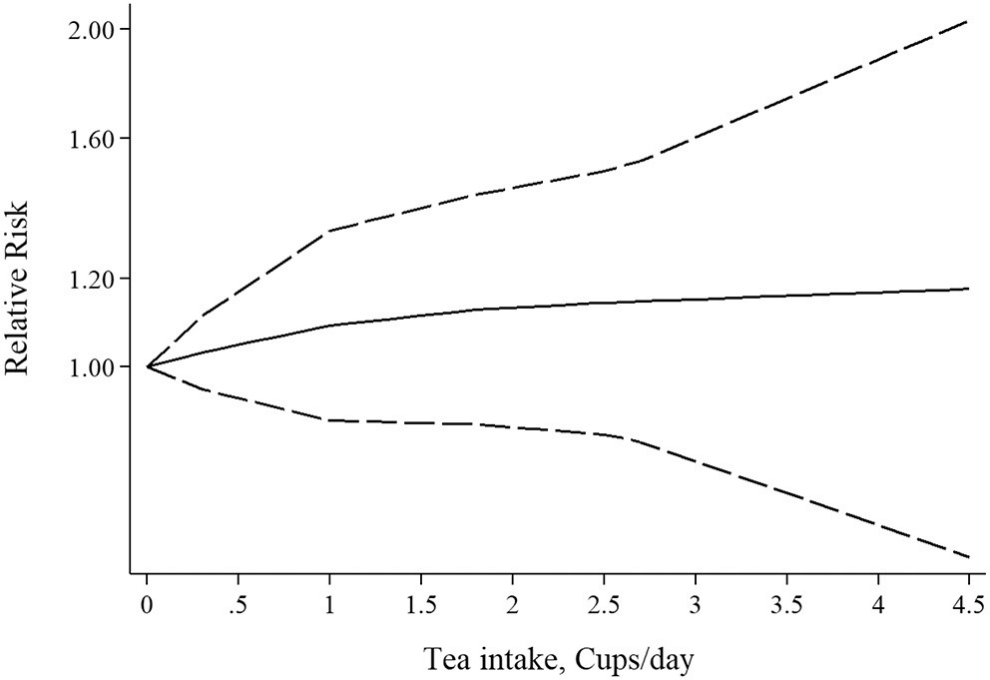
Non-linear dose-response association of tea intake and risk of rheumatoid arthritis.

Three studies had examined the association between tea intake and risk of RA (39–41). Pooled effect sizes for the highest vs. lowest category of tea intake showed no significant association between tea intake and risk of RA (RR: 1.05; 95% CI: 0.73–1.53; Figure 5A), with significant heterogeneity among the studies (*I*^2^ = 69.1%; *P* = 0.03). Furthermore, sensitivity analysis found that the overall effect size did not depend on a single study. There was no evidence of publication bias by Begg's test (*P* = 0.60) and Egger's test (*P* = 0.70). There was no statistically significant association between one cup of tea per day and risk of RA (RR: 1.04; 95% CI: 0.94–1.16; Figure 5B). Also, non-linear dose-response analysis did not reveal any association (P_non−linearity_ = 0.61; Figure 6). Using the NutriGrade score, the quality of the evidence was found to be low (Supplementary Table 4).

## Discussion

RA is a chronic autoimmune inflammatory disease that leads to inflammation and joint pain that might affect other organs. Dietary components can affect the severity or improvement of the disease symptoms. Numerous studies have shown that coffee and tea consumption could increase inflammatory markers in these patients (20, 38, 41). On the other hand, a number of other studies have revealed the protective role of coffee and tea in RA (18, 19). As a result, we conducted a systematic review and meta-analysis on relevant cohorts to summarize the findings of these studies. The results of our systematic review and meta-analysis of five cohort studies showed a significant association between coffee intake and the risk of RA. We found a similar positive association between decaffeinated coffee intake and the risk of RA. We did not observe significant associations between caffeine, caffeinated coffee and tea intake with the risk of RA.

In the present study, we found that higher coffee intake was associated with an increased risk of RA, which is consistent with the case-control study by Pedersen et al. who found a significant, positive association between coffee intake and the risk of RA (20). The possible mechanism by which coffee may associated with the increased risk of RA is related to the effect of coffee on the level of inflammatory markers. Zampelas et al., in a cohort study, found that IL-6 increased among those drinking more than four cups per day (28). However, there are studies that report conflicting results. Some studies have shown that coffee can exert anti-inflammatory and antioxidant effects due to compounds such as caffeine, cafestol, chlorogenic acid and trigonelline (43). This theory has been supported in some observational studies. The Nurses' Health Study II showed a negative association between coffee consumption and c-reactive protein, interleukin-6, tumor necrosis factor alpha and other inflammatory markers (44).

Another mechanism for significant association between coffee intake and an increased risk of RA may be the lack of controlling for confounding variables such as physical activity, smoking, and other lifestyle-related factors. Studies have shown that people who consume higher amounts of coffee are more likely to be a smoker (45, 46). Smoking is a risk factor for increased levels of inflammation and diseases such as RA (47). Our findings revealed that increased caffeine consumption was not related to an increase in risk of RA. Thus, besides caffeine, other chemical ingredients that are used in the growing or preparation of coffee may be responsible for this finding.

With regards to caffeinated coffee, we found no significant association between its intake and the risk of RA. Some studies have demonstrated that people who drank caffeinated coffee had higher levels of IL-6 compared to those consuming no coffee (48, 49). On the other hand, Wedick et al., in a clinical trial, have shown that caffeinated coffee can increase adiponectin levels (49). Some studies have pointed out that adiponectin can play a pro-inflammatory role in the pathophysiology of autoimmune disease such as RA through stimulating the secretion of inflammatory mediators (50, 51).

We also found a positive association between decaffeinated coffee consumption and the risk of RA. The mechanism involved seems to be the method of extracting caffeine from coffee by direct application of industrial solvents including benzene, acetone, ammonium hydroxide, sulfuric acid, ethyl acetate, methylene chloride, chloroform, ether, alcohol, trichloroethylene and carbon tetrachloride (52). Chronic ingestion of solvent residues, even small quantities, could result in connective tissue disorders such as scleroderma, lupus, and RA (53). Additionally, decaffeinated coffee has a smaller even higher antioxidant activity than regular coffee (54, 55).

In our study, there was no significant relationship between tea consumption and the risk of RA. However, a case-control study in Iran showed an inverse association between tea consumption and the risk of RA (18). Similarly, Jin et al., in a cross-sectional study on RA patients, found an inverse association between high tea consumption (>750 mL/day) and disease severity in patients with RA (56). A number of studies have also shown that tea consumption could have a beneficial effect on inflammatory factors which is due to catechins and other flavonoids (57, 58). The null findings observed in our study may be due to the insufficient prospective cohort studies examining the association between tea intake and the incidence of inflammation and RA. Also, the included studies did not report the risk of RA according to the type of tea. Although the exact role of different types of tea in the pathogenesis of RA remains unknown, some publications have indicated that green tea has an immunomodulatory effect and, thus, can protect against RA (59). Furthermore, Rambod et al., in a case-control study, showed that green tea intake was correlated with a 35% decreased risk of RA (19). On the other hand, there are studies that have indicated that black tea intake was not associated with the level of inflammatory markers (60–62). Given that all types of tea are processed differently, this may lead to changes in chemical ingredients and finally different properties (40).

We did not observe a significant relationship between caffeine intake and the risk of RA. Our finding is in agreement with a previous systematic review on clinical trials indicating that caffeine has no definite role in short-term inflammatory responses and these pathways are not clearly understood (63). However, a cross-sectional study found a statistically significant reduction in disease activity and cytokine levels in systemic lupus erythematosus patients who had higher intakes of caffeine (64). It was shown that caffeine metabolites, such as xanthine and theobromine, have antioxidant activities and decrease hydroxyl radicals (65). Also, it is believed that caffeine has an adenosine antagonist effect and can induce a decrease in cytokine production (66).

The present study has some strengths that should be acknowledged. Unlike previous meta-analyses, we performed a dose-response analysis which has provided more precise results than the highest vs. lowest analysis. Also, we assessed the association of different types of coffee (caffeinated vs. decaffeinated) with RA which revealed their different effects on RA. Moreover, the included studies were a cohort with a relatively large sample size which increased the generalizability of the findings and statistical inferences.

In spite of these strengths, this study has some limitations which should be taken into account when interpreting the results. First, our study included a small number of publications that weakened the interpretation of the relationships in the meta-analyses. Second, the results are subject to residual confounding because of the cohort design of the included studies. Third, most of the included studies used a food frequency questionnaire to evaluate dietary intake and, therefore, measurement error and misclassification of the study participants in terms of exposure were inevitable. Finally, the size of the cup of coffee and tea in the entire study was not determined, which could influence the observed associations.

## Conclusion

Subject to the limitations such as low number of studies and weak statistical power, we observed a positive association between coffee, decaffeinated coffee intake and the risk of RA. However, no statistically significant associations were observed between caffeinated coffee, caffeine or tea consumption with the incidence of RA. More experimental and observational studies are needed to substantiate such findings.

## Data Availability Statement

The original contributions presented in the study are included in the article/Supplementary Materials, further inquiries can be directed to the corresponding author.

## Author Contributions

FA, FD, and AH contributed to the conception, design literature search, and manuscript drafting. AJ and AF contributed to the design, statistical analyses, and interpretation of data. HM contributed to the conception, design, data interpretation, drafting of the manuscript, and supervised the study. Final manuscript was approved by all authors approved prior to submission.

## Conflict of Interest

The authors declare that the research was conducted in the absence of any commercial or financial relationships that could be construed as a potential conflict of interest.

## Publisher's Note

All claims expressed in this article are solely those of the authors and do not necessarily represent those of their affiliated organizations, or those of the publisher, the editors and the reviewers. Any product that may be evaluated in this article, or claim that may be made by its manufacturer, is not guaranteed or endorsed by the publisher.
