# Supplementary material for: Caffeine, Coffee, Tea and Risk of Rheumatoid Arthritis: Systematic Review and Dose-Response Meta-analysis of Prospective Cohort Studies
Source: Front Nutr. 2022 Feb 10;9:822557. doi: 10.3389/fnut.2022.822557 (PMC8866764; doi:10.3389/fnut.2022.822557)
Supplement: Supplementary file 1 [file Table_1.DOCX]

**MOOSE Checklist**

From: Stroup DF, Berlin JA, Morton SC, et al (2000) Meta-analysis of observational studies in epidemiology: A proposal for reporting. JAMA 283:2008–2012. doi:10.1001/jama.283.15.2008.

|  | Reported on page | Comments |
| --- | --- | --- |
| **Reporting of background should include** | | |
| Problem definition | 3 |  |
| Hypothesis statement | 3 |  |
| Description of study outcome(s) | 3 |  |
| Type of exposure or intervention used | 3 |  |
| Type of study designs used | 4 |  |
| Study population | 4 |  |
| **Reporting of search strategy should include** | | |
| Qualifications of searchers (e.g. librarians and investigators) | 4 | The qualifications of searchers are reported in the protocol, which is free available at PROSPERO. |
| Search strategy, including time period used in the synthesis and key words | 4-5 and supplementary table 1 |  |
| Effort to include all available studies, including contact with authors | 4-5 |  |
| Databases and registries searched | 4 |  |
| Search software used, name and version, including special features used (e.g. explosion) | 4 |  |
| Use of hand searching (e.g. reference lists of obtained articles) | 5 |  |
| List of citations located and those excluded, including justification |  | The PRISMA flow chart describes the process of the literature search process. The citation list of excluded articles is available upon request |
| Method of addressing articles published in languages other than English |  | Our search strategy did not identify any article published in languages other than English |
| Method of handling abstracts and unpublished studies | 4 |  |
| Description of any contact with authors | 5 |  |
| **Reporting of methods should include** | | |
| Description of relevance or appropriateness of studies assembled for assessing the hypothesis to be tested | 5 |  |
| Rationale for the selection and coding of data (e.g. sound clinical principles or convenience) | 5 |  |
| Documentation of how data were classified and coded (e.g. multiple raters, blinding and interrater reliability) | 5 |  |
| Assessment of confounding (e.g. comparability of cases and controls in studies where appropriate) | NA |  |
| Assessment of study quality, including blinding of quality assessors, stratification or regression on possible predictors of study results | NA |  |
| Assessment of heterogeneity | 6 | inter-study heterogeneity was assessed using the Cochran Q statistic and quantified using the I2 statistic, where I2>50% at PQ<0.10 was considered evidence of substantial heterogeneity |
| Description of statistical methods (e.g. complete description of fixed or random effects models, justification of whether the chosen models account for predictors of study results, dose-response models, or cumulative meta-analysis) in sufficient detail to be replicated | 6-7 |  |
| Provision of appropriate tables and graphics | Tables 1and supplementary file |  |
| **Reporting of results should include** | | |
| Graphic summarizing individual study estimates and overall estimate | Figure 2 and Figure 5 |  |
| Table giving descriptive information for each study included | Table 1 |  |
| Results of sensitivity testing (e.g. subgroup analysis) | 8-10 |  |
| Indication of statistical uncertainty of findings | 8-10 |  |
| **Reporting of discussion should include** | | |
| Quantitative assessment of bias (e.g. publication bias) | 8-10 |  |
| Justification for exclusion (e.g. exclusion of non-English language citations) | 5 | Exclusion criteria are stated in material and methods section |
| Assessment of quality of included studies | 7 |  |
| **Reporting of conclusions should include** | | |
| Consideration of alternative explanations for observed results | 11-14 |  |
| Generalization of the conclusions (i.e. appropriate for the data presented and within the domain of the literature review) | 14 |  |
| Guidelines for future research | 14 |  |
| Disclosure of funding source | 15 |  |
