# Supplementary material for: Caffeine, Coffee, Tea and Risk of Rheumatoid Arthritis: Systematic Review and Dose-Response Meta-analysis of Prospective Cohort Studies
Source: Front Nutr. 2022 Feb 10;9:822557. doi: 10.3389/fnut.2022.822557 (PMC8866764; doi:10.3389/fnut.2022.822557)
Supplement: Supplementary file 2 [file Data_Sheet_1.docx]

| **Database**  **Supplemental Table 1.** Search strategies including the key terms and the queries for each database. | **key terms and the queries** |
| --- | --- |
| PubMed | #1**"Arthritis, Rheumatoid"[Mesh] OR rheumatoid[Title/Abstract] OR "rheumatoid arthritis"[Title/Abstract] OR rheumatoid[Title/Abstract]**  **#**2 "Camellia sinensis"[Mesh] OR "Tea"[Mesh] OR "Coffee"[Mesh] OR "Camellia sinensis"[Title/Abstract] OR Tea[Title/Abstract] OR "Coffee"[Title/Abstract]"Caffeine"[Title/Abstract]  **#1 and #2** |
| Scopus | #1 TITLE-ABS-KEY (“rheumatoid”) OR TITLE-ABS-KEY (“rheumatoid arthritis”)  #2 TITLE-ABS-KEY ("Camellia sinensis") OR TITLE-ABS-KEY(Tea) OR TITLE-ABS-KEY(Coffee) OR TITLE-ABS-KEY(Caffeine)  #1 and #2 |
| Embase | #1 'rheumatoid':ti,ab,kw OR 'rheumatoid arthritis':ti,ab,kw  #2 ' tea':ti,ab,kw OR 'green tea extract':ti,ab,kw OR 'camellia sinensis':ti,ab,kw OR ' coffee':ti,ab,kw OR 'green tea':ti,ab,kw OR 'green tea':ti,ab,kw OR 'black tea':ti,ab,kw OR 'caffeine':ti,ab,kw  #1 and #2 |

**Supplementary Table 2.** PICOS criteria for inclusion and exclusion of studies

|  | **Inclusion criteria** | **Exclusion criteria** |
| --- | --- | --- |
| **Population**  **Intervention / Exposure**  **Comparison**  **Outcomes**  **Study type** | General population of adults  Dietary intakes of Tea or Coffee or Caffeinated coffee or Decaffeinated coffee or Caffeine  Odds ratio, rate or risk ratios, relative risk (RRs), or hazard ratios  Risk of rheumatoid arthritis  Prospective cohort studies | Aged <18 years  Dietary intakes of other caffeinated beverages  Other risk estimates  Risk of other inflammatory disease  Cross-sectional, case-control, ecological, retrospective observational studies, clinical trials, and non-human studies |

| **Supplemental Table 3**: Reason for exclusion of retrieved articles | |
| --- | --- |
| References | Reason for exclusion |
| 1. Marchand, N., et al. (2019). "A healthy plant-rich diet and the risk of rheumatoid arthritis in women." Arthritis and Rheumatology **71**: 289-290. | No relevant exposure reported |
| 1. Prescha, A., et al. (2018). "Diet quality and its relationship with antioxidant status in patients with rheumatoid arthritis." Oxidative Medicine and Cellular Longevity **2018**. | No relevant outcome reported |
| 1. Malaviya, A. N. (2017). "Methotrexate intolerance in the treatment of rheumatoid arthritis (RA): effect of adding caffeine to the management regimen." Clinical Rheumatology **36**(2): 279-285. | No relevant outcome reported |
| 1. Macejová, Ž. and M. Macej (2011). "Epidemiology of rheumatoid arthritis - Risk factors, incidence, prevalence, duration of disease." Rheumatologia **25**(2): 85-89. | Review |
| 1. Kunsch, C., et al. (2005). "Oxidative stress and the use of antioxidants for the treatment of rheumatoid arthritis." Current Medicinal Chemistry: Immunology, Endocrine and Metabolic Agents **5**(3): 249-258. | Review |
| 1. Bae, S. C. and Y. H. Lee (2018). "Coffee consumption and the risk of rheumatoid arthritis and systemic lupus erythematosus: a Mendelian randomization study." Clinical Rheumatology **37**(10): 2875-2879. | Irrelevant study design |
| 1. Mosalmanzadeh, N., et al. (2020). "Major dietary patterns and food groups in relation to rheumatoid arthritis in newly diagnosed patients." Food Science and Nutrition. | Irrelevant study design |
| 1. Li, J., et al. (2019). "The association of tea consumption with the disease activity of rheumatoid arthritis." European Journal of Immunology **49**: 926. | Irrelevant study design |
| 1. Rambod, M., et al. (2018). "The impact of dietary habits on the pathogenesis of rheumatoid arthritis: a case-control study." Clinical Rheumatology **37**(10): 2643-2648. | Irrelevant study design |
| 1. Chamizo-Carmona, E., et al. (2017). "The effect of smoking, alcohol and caffeine on early rheumatoid arthritis outcomes." Annals of the Rheumatic Diseases **76**: 1121-1122. | Irrelevant study design |
| 1. Yoshimura, H., et al. (2019). "Differences of lifestyle habits of smoking, drinking alcohol and caffeinated coffee consumption between rheumatoid arthritis patients and healthy control-tomorrow study." Annals of the Rheumatic Diseases **78**: 645. | Irrelevant study design |

**Supplementary Table 4**. Scoring for the different components of NutriGrade for each outcome.

| **Exposure** | **Outcome** | **Risk of bias**^1^ | **Precision**^2^ | **Heterogeneity**^3^ | **Directness**^4^ | **Publication bias**^5^ | **Funding bias**^6^ | **Effect size**^7^ | **Dose-response**^8^ | **Sum** | **NutriGrade** |
| --- | --- | --- | --- | --- | --- | --- | --- | --- | --- | --- | --- |
| Coffee | RA | 2 | 0 | 0 | 1 | 0 | 1 | 1 | 1 | 6 | Moderate |
| Caffeinated coffee | RA | 2 | 0 | 0 | 1 | 0 | 1 | 0 | 0 | 4 | Low |
| De-caffeinated coffee | RA | 2 | 0 | 0 | 0 | 0 | 1 | 1 | 1 | 5 | Moderate |
| Tea | RA | 2 | 0 | 0 | 1 | 0 | 1 | 0 | 0 | 4 | Low |
| Caffeine | RA | 2 | 0 | 0 | 1 | 0 | 1 | 0 | 0 | 4 | Low |
| ^1^ Modifications of original NutriGrade-scoring system: Study quality Newcastle Ottawa Scale: ≥8 (2 points); ≥7-<8 (1.5 points), ≥6-<7(1 point).  ^2^ >5000 cases and the 95%CI excludes the null value (1 point), <5000 cases or >5000 cases but 95%CI includes the null value (0 point).  ^3^ 0 to 1 point on the basis of *I*^2^.  ^4^ No important differences in the population or hard clinical outcomes (1 point), important differences in the population (0 point).  ^5^ <5 studies or sever evidence of bias or publication bias not assessed (0 point), no evidence of bias for 5-9 studies or moderate evidence of bias for ≥10 studies (0.5 point), no evidence of bias for ≥10 studies (1 point).  ^6^ Funded by academic institutions or research institutions (1 point), funded by private institutions, foundations, or nongovernmental organizations (0.5 point), Industry funding or conflict of interest (0 point).  ^7^ No effect (RR: 0.80–1.20) when comparing the highest vs. lowest category (0 point), moderate effect size (RR: <0.80–0.50 and >1.20–2, and corresponding test is statistically significant) when comparing the highest vs. lowest category (1 point), large effect size (RR: <0.50 and >2.00, and corresponding test is statistically significant) when comparing the highest vs. lowest category (2 point).  ^8^ No dose-response analysis or dose-response analysis with corresponding statistical test no significant (0 point), significant linear or nonlinear dose-response relationship (1 point). | | | | | | | | | | | |
